# What Drives Anticoagulant Selection in Patients Aged ≥85 Years with Atrial Fibrillation? Insights from the CRAFT Registry

**DOI:** 10.3390/jcm15103806

**Published:** 2026-05-15

**Authors:** Janusz Bednarski, Monika Szewczak, Marta Grzesiak, Emilia Kamińska, Maciej Karczewski, Karolina Własiuk, Michał Wojewódzki

**Affiliations:** 1 Department of Cardiology, St. John Paul II Western Hospital, 05-825 Grodzisk Mazowiecki, Poland; monika.szewczak@onet.pl (M.S.); martagrzesiak33@gmail.com (M.G.); egzkaminska@gmail.com (E.K.); maciekkarczewski111@gmail.com (M.K.); karolina.wlasiuk@onet.pl (K.W.); michaljerzywojewodzki@gmail.com (M.W.); 2 Clinic of Cardiology, Lazarski University, Swieradowska Str. 43, 02-662 Warsaw, Poland

**Keywords:** atrial fibrillation, anticoagulation, elderly, direct oral anticoagulants, stroke prevention, renal function, registry

## Abstract

**Background**: Anticoagulation management in very elderly patients with atrial fibrillation (AF) is particularly challenging due to the coexistence of high thromboembolic and bleeding risks, often compounded by multiple comorbidities. Randomized clinical trials rarely include patients aged ≥85 years, leaving important gaps in our understanding of how anticoagulant therapies are selected in this growing population. **Methods**: We analyzed data from the CRAFT registry, including 2914 patients hospitalized with AF. Patients were stratified into two age groups: <85 years (*n* = 2322) and ≥85 years (*n* = 592). Baseline clinical characteristics, comorbidities, and laboratory parameters were compared between groups. Separate multivariable logistic regression analyses were performed for each age group to identify independent predictors of anticoagulant therapy selection. **Results**: Patients aged ≥85 years exhibited a distinct clinical profile, characterized by higher thromboembolic risk and a greater prevalence of heart failure, renal dysfunction, anemia, and structural heart disease. Renal function was significantly impaired (median eGFR 47.6 vs. 60.0 mL/min; *p* < 0.001), while NT-proBNP levels were higher and hemoglobin levels lower in this group. Multivariable analysis revealed clear age-related differences in determinants of treatment selection. In patients < 85 years, anticoagulant choice was influenced by multiple clinical factors, including CHA_2_DS_2_-VA score, renal function, bleeding risk, coronary artery disease, and prior revascularization. In contrast, in patients ≥ 85 years, only two independent predictors remained significant: thromboembolic risk (CHA_2_DS_2_-VA score; OR 1.34, 95% CI 1.11–1.64) and renal function (eGFR; OR 0.64, 95% CI 0.47–0.89). Anticoagulation in this group was predominantly based on reduced-dose DOACs, with apixaban used most frequently. **Conclusions**: Very elderly patients with AF represent a clinically distinct, high-risk population. While anticoagulant selection in younger elderly patients reflects a multifactorial decision process, treatment in those aged ≥85 years appears to rely primarily on thromboembolic risk and renal function, suggesting a more streamlined—and potentially oversimplified—approach.

## 1. Introduction

In Poland, as in other European countries, the population aged ≥85 years is steadily increasing, with projections suggesting a doubling by 2050 [1]. Current estimates indicate that approximately 840,000 individuals in Poland fall within this age group [2]. Atrial fibrillation (AF) is particularly prevalent among the elderly, affecting an estimated 15–25% of individuals aged ≥85 years [3,4,5,6]. Data from the NOMED-AF study, which used 30-day electrocardiographic monitoring, reported an even higher prevalence of 31.9% in this population [7,8].

If these estimates hold, this would correspond to approximately 270,000 very elderly individuals with AF in Poland and nearly 4 million across the European Union. Patients in this age group frequently present with multiple comorbidities, including dementia, chronic kidney disease, hypertension, diabetes, and an increased risk of falls, all of which complicate clinical management.

Advancing age is associated with both increased thromboembolic and bleeding risk, as reflected by higher CHA_2_DS_2_-VA and HAS-BLED scores. Age-related physiological changes—such as altered coagulation factor levels, impaired fibrinolysis, endothelial dysfunction, and reduced mobility—further promote thrombus formation [9,10,11,12]. In addition, age-related organ dysfunction, particularly involving the kidneys and liver, along with changes in body composition and polypharmacy, significantly alter drug pharmacokinetics and pharmacodynamics. These factors collectively increase the risk of adverse drug reactions, including bleeding complications.

Consequently, anticoagulation therapy in very elderly patients with AF remains particularly challenging, requiring a careful balance between the benefits of stroke prevention and the increased risk of bleeding. Despite the rapidly growing number of patients aged ≥85 years, evidence to guide treatment decisions in this population is still limited because very elderly individuals are rarely well-represented in randomized clinical trials of direct oral anticoagulants. At the same time, the ≥85-year threshold is commonly used in AF registries and observational studies to define the “oldest-old” population, facilitating comparisons between studies and highlighting the specific clinical challenges associated with very advanced age. Nevertheless, real-world data on factors influencing anticoagulant selection in these patients remain limited.

Therefore, the aim of this study was to evaluate the clinical characteristics and determinants of anticoagulant therapy in patients with AF, with a particular focus on those aged ≥85 years. Using data from the CRAFT registry, we sought to identify the key clinical factors influencing treatment decisions and to determine whether these differ between younger elderly patients and those of very advanced age.

## 2. Materials and Methods

### 2.1. Study Design

This study was based on data from the CRAFT registry (MultiCenter expeRience in AFib patients Treated with OAC), registered at ClinicalTrials.gov (Bethesda, MA, USA) (NCT02987062). The registry comprises a retrospective analysis of hospital discharge records for patients with AF treated with oral anticoagulants, including vitamin K antagonists (VKAs) and direct oral anticoagulants (DOACs).

The study was based on retrospective analysis of anonymized registry data and did not involve any intervention or direct patient contact. According to national regulations governing non-interventional retrospective studies using anonymized data, formal Institutional Review Board approval was not required. The registry initially included all adult patients hospitalized with AF at an academic center and a regional hospital between 2011 and 2016. Patients receiving anticoagulation for indications other than AF—such as venous thromboembolism or mechanical heart valves—were excluded.

The registry collected data on demographics, comorbidities, AF type (paroxysmal, persistent, or permanent), selected laboratory parameters, and treatment details. Since 2017, the registry has been maintained exclusively at the regional hospital, and the present analysis is based on these data. The design, objectives, and selected findings of the registry have been reported previously [13,14,15].

### 2.2. Statistical Analysis

Statistical analyses were performed using SPSS software version 26.0 (IBM Corp., Armonk, NY, USA).

Continuous variables were summarized using mean (*M*), median (*Md*), standard deviation (*SD*), and sample size (*N*), and group comparisons were conducted using the Mann–Whitney U test due to non-normal data distribution.

Categorical variables were expressed as counts and percentages and compared using Pearson’s chi-square (χ^2^) test to assess associations between age groups (<85 vs. ≥85 years), clinical characteristics, comorbidities, and treatment patterns.

Logistic regression analysis was used to identify clinical factors independently associated with antithrombotic treatment strategies. Associations were expressed as odds ratios (ORs) with corresponding confidence intervals. Model performance was evaluated using −2 log-likelihood, the chi-square test, and pseudo-R^2^ statistics (Cox and Snell, Nagelkerke). A *p*-value <0.05 was considered statistically significant.

Separate logistic regression models were constructed for each anticoagulant regimen, defined as a specific drug–dose combination (e.g., apixaban 2.5 mg, rivaroxaban 20 mg). Thus, the dependent variable in each model was the prescription of a given anticoagulant strategy.

Due to the registry’s retrospective nature, some variables had limited missing data. Cases with incomplete data for variables included in a given regression model were excluded from that specific analysis using a complete-case approach.

Due to limited variability in some treatment categories in the ≥85 years group, regression models could not be constructed for all therapies. Only successfully developed models are presented.

## 3. Results

### 3.1. Baseline Characteristics

The study included 2914 patients with AF, of whom 2322 (79.7%) were <85 years and 592 (20.3%) were ≥85 years. In the older group, the mean age was 88.43 ± 2.99 years. Most patients were aged 85–89 years (*n* = 403), followed by 90–94 years (*n* = 162) and ≥95 years (*n* = 27).

**Figure 1:**
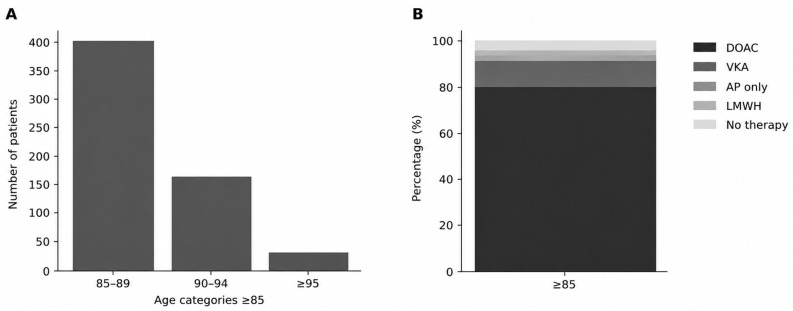
Characteristics of patients aged ≥85 years. (**A**) Age distribution within the ≥85 years group, with the majority of patients aged 85–89 years and fewer in the older subgroups. (**B**) Antithrombotic treatment patterns in patients aged ≥85 years. Direct oral anticoagulants were the predominant therapy, followed by vitamin K antagonists, while other treatment strategies were infrequent.

In patients aged ≥85 years, direct oral anticoagulants (DOACs) were the most commonly used treatment (480 patients, 81.1%), followed by vitamin K antagonists (63 patients, 10.6%). Other treatment strategies included no anticoagulation (28 patients, 4.7%), low-molecular-weight heparin (11 patients, 1.9%), and antiplatelet therapy alone (10 patients, 1.7%) (Figure 1A,B).

A marked difference in sex distribution was observed, with a higher proportion of women in the ≥85 years group (62.8% vs. 39.5%; χ^2^ = 102.89, *p* < 0.001). Patients aged ≥85 years had higher CHA_2_DS_2_-VA scores than younger patients (4.60 vs. 3.77; *p* < 0.001). Permanent AF was the predominant subtype (58.7%).

Comorbidities were common and included heart failure (73.3%), atherosclerosis (51.2%), and valvular heart disease (41.8%). Diabetes mellitus was present in 28.9% of patients, and 15.5% had a history of myocardial infarction. Bleeding or anemia was reported in 29.1% of patients.

Renal dysfunction was highly prevalent in the ≥85 years group: 316 patients (53.4%) had an eGFR < 50 mL/min, including 82 patients (13.9%) with severe renal impairment (eGFR < 30 mL/min).

**Figure 2:**
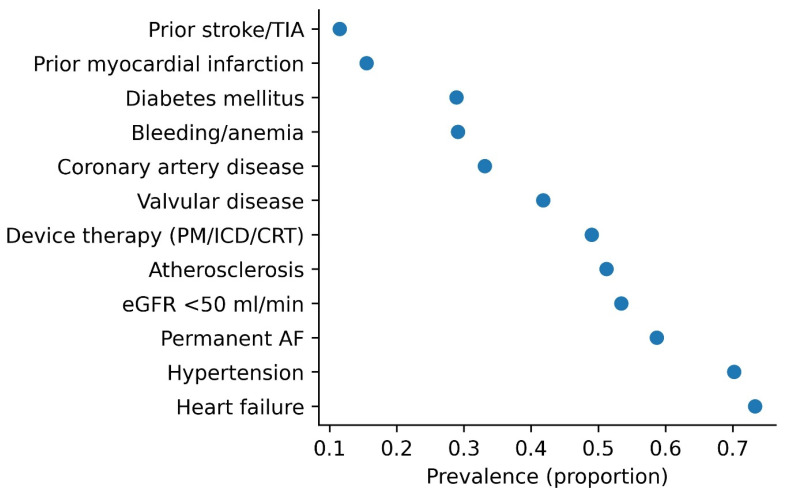
Clinical profile of patients aged ≥85 years with atrial fibrillation. Values represent the prevalence of selected clinical characteristics. Abbreviations: AF, atrial fibrillation; eGFR, estimated glomerular filtration rate; PM, pacemaker; ICD, implantable cardioverter–defibrillator; CRT, cardiac resynchronization therapy; TIA, transient ischemic attack.

**Figure 3:**
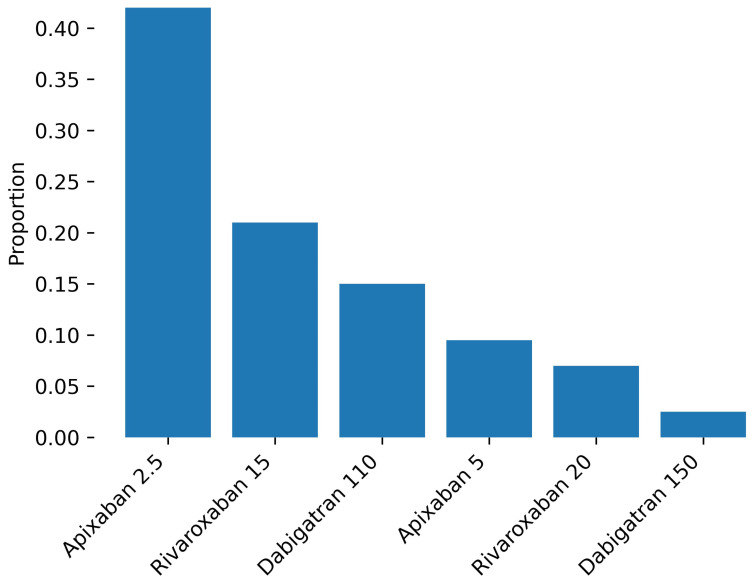
Distribution of anticoagulant therapies in the study population. Reduced-dose regimens predominated, with apixaban 2.5 mg being the most commonly used, followed by rivaroxaban 15 mg and dabigatran 110 mg. Full-dose therapies were less frequently prescribed.

Baseline characteristics stratified by age are summarized in Table 1 and Figure 2. The distribution of specific DOAC regimens is presented in Figure 3.

### 3.2. Multivariable Logistic Regression

Multivariable logistic regression in patients aged <85 years showed that treatment decisions were influenced by multiple clinical factors, including CHA_2_DS_2_-VA, renal function, coronary artery disease, PCI, and bleeding risk. Detailed model characteristics are presented in Supplementary Table S2.

In patients aged ≥85 years, only two logistic regression models reached statistical significance, likely reflecting both the smaller sample size and more uniform treatment patterns in this group (Table 2). The administration of reduced-dose apixaban (2.5 mg) was associated with a higher CHA_2_DS_2_-VA score and lower eGFR, indicating that greater thromboembolic risk and impaired renal function increased the likelihood of its prescription. In contrast, higher CHA_2_DS_2_-VA scores were associated with a lower probability of receiving dabigatran 150 mg, suggesting a cautious approach to full-dose therapy in very elderly patients with higher thromboembolic risk.

## 4. Discussion

This study provides insight into real-world anticoagulation practice in patients aged ≥85 years with atrial fibrillation (AF)—a rapidly expanding and clinically vulnerable population that remains underrepresented in randomized trials. Several findings merit particular attention.

First, patients aged ≥85 years in our cohort exhibited a markedly higher thromboembolic risk, along with a greater burden of comorbidities, including heart failure, structural heart disease, and renal dysfunction. The relatively preserved left ventricular ejection fraction observed in the ≥85 years group likely reflects the predominance of heart failure with preserved ejection fraction, which is common in very elderly patients with atrial fibrillation. This profile is in line with observations from large registries such as Fushimi AF [16], ANAFIE [17], and GARFIELD-AF [18,19], which consistently describe very elderly patients as a high-risk group with a more adverse clinical profile and worse outcomes. Our data reinforce the view that age should be understood not simply as a demographic characteristic, but as a composite marker of cumulative clinical risk in AF.

Importantly, the coexistence of elevated thromboembolic and bleeding risks highlights the fundamental therapeutic dilemma in this population. Anticoagulation decisions in patients aged ≥85 years are rarely straightforward; rather, they involve a continuous balancing of competing risks and inevitably rely on clinical judgment. The consistency of our findings with international data suggests that this high-risk phenotype is largely independent of geographic setting or healthcare system.

Second, we observed a clear predominance of reduced-dose DOACs, with apixaban 2.5 mg accounting for a substantial proportion of prescriptions, while full-dose regimens were used infrequently. This pattern mirrors real-world data from studies such as ELDERCARE-AF [20], Fushimi AF [16], ANAFIE [17], POL-AF [21], FRAIL-AF [22], and GLORIA-AF [23]. However, it also raises important questions regarding the appropriateness of dose reduction in very elderly patients.

Although dose reduction is often justified by impaired renal function or concerns about bleeding, growing evidence suggests that underdosing may be common in routine practice. Our findings likely reflect a cautious, risk-averse approach in which clinicians prioritize bleeding avoidance, potentially at the expense of optimal thromboembolic protection. At the same time, the widespread use of DOACs in our cohort is consistent with data from COMBINE-AF [24] and other studies demonstrating a favorable risk–benefit profile of these agents across age groups.

Third, and perhaps most strikingly, treatment allocation in our study appeared to be driven primarily by two factors: thromboembolic risk (CHA_2_DS_2_-VA score) and renal function (eGFR). The inability to construct robust multivariable models—due to limited variability in clinical decision-making—represents an important finding in itself. It suggests that therapeutic strategies for this age group may be either highly standardized or overly simplified.

While the central role of CHA_2_DS_2_-VA and renal function is well-established, the apparent underweighting of other clinically relevant factors—such as frailty, functional status, polypharmacy, or prior bleeding—raises concerns as to whether current decision-making fully reflects the complexity of very elderly patients. Although similar determinants have been described in studies such as ENGAGE AF–TIMI 48 [25], few analyses have specifically addressed the potential narrowing of decision frameworks in the oldest-old population.

Taken together, our findings support the concept of a “restricted therapeutic landscape” in patients aged ≥85 years, where treatment decisions are based on a relatively limited set of parameters. While this approach may improve feasibility and perceived safety in everyday practice, it may also point to a gap between guideline recommendations and real-world clinical reasoning. A comprehensive comparison of anticoagulation patterns across major registries and trials is provided in Supplementary Table S1.

In summary, the CRAFT study confirms that very elderly patients with AF constitute a distinct high-risk population characterized by multimorbidity, renal dysfunction, and increased thromboembolic risk. In this group, anticoagulation strategies are dominated by reduced-dose DOACs, and treatment decisions appear to rely primarily on a narrow set of clinical indicators. Compared with other cohorts, the CRAFT population showed a particularly high rate of anticoagulation use (91.7%), a strong predominance of DOAC therapy, frequent use of apixaban, and a substantial burden of heart failure and renal impairment.

## 5. Conclusions

Very elderly patients with atrial fibrillation represent a distinct, high-risk population characterized by a substantial burden of comorbidities, impaired renal function, and increased thromboembolic risk. In this group, anticoagulation strategies are largely based on reduced-dose direct oral anticoagulants, with clinical decision-making primarily guided by thromboembolic risk and renal function.

The high rate of anticoagulation use observed in the CRAFT cohort—predominantly with DOACs, particularly apixaban—reflects a pragmatic, safety-oriented approach in routine practice. However, these findings also suggest that current treatment strategies may be overly simplified and may not fully capture the complexity of this population. More nuanced, individualized, and evidence-based approaches are needed to optimize care in very elderly patients with AF.

## 6. Study Limitations

This study has several limitations. First, it is based on registry data from a single healthcare setting, which may limit the generalizability of the findings to other institutions or populations. Second, although the overall cohort was large, the number of very elderly patients was relatively smaller compared with younger groups, which may have reduced the statistical power to detect certain associations in this subgroup.

Third, because this is an observational study, causal relationships between clinical characteristics and treatment decisions cannot be established. Treatment choices may have been influenced by factors not captured in the registry, such as frailty, cognitive status, patient preferences, or other individualized considerations. In addition, complete HAS-BLED score calculation was not consistently available for all patients due to limitations inherent to retrospective registry data collection. Therefore, individual bleeding-related variables, including prior bleeding/anemia and renal dysfunction, were analyzed instead. Finally, detailed outcome analyses, such as bleeding events or long-term thromboembolic risk, were not included. Future studies should address these aspects to provide a more comprehensive understanding of anticoagulation management in this population.

## Figures and Tables

**Table 1:** Baseline Clinical and Laboratory Characteristics According to Age.

Variable	<85 Years (*n* = 2322)	≥85 Years (*n* = 592)	Total (*n* = 2914)	*p*-Value
Clinical characteristics				
Sex (female)	918 (39.5%)	372 (62.8%)	1290 (44.3%)	<0.001
Sex (male)	1404 (60.5%)	220 (37.2%)	1624 (55.7%)	
CHA_2_DS_2_-VA (mean ± SD)	3.77 ± 1.65	4.60 ± 1.25	—	<0.001
CHA_2_DS_2_-VA score 0	61 (2.6%)	0 (0.0%)	61	<0.001
CHA_2_DS_2_-VA score 1	144 (6.2%)	0 (0.0%)	144	
CHA_2_DS_2_-VA score 2	318 (13.7%)	28 (4.7%)	346	
CHA_2_DS_2_-VA score 3	449 (19.3%)	82 (13.8%)	531	
CHA_2_DS_2_-VA score 4	560 (24.1%)	166 (28.0%)	726	
CHA_2_DS_2_-VA score 5	478 (20.6%)	192 (32.4%)	670	
CHA_2_DS_2_-VA score 6	205 (8.8%)	87 (14.7%)	292	
CHA_2_DS_2_-VA score 7	75 (3.2%)	28 (4.7%)	103	
CHA_2_DS_2_-VA score 8	31 (1.3%)	10 (1.7%)	41	
Permanent AF	871 (37.7%)	343 (58.7%)	1214	<0.001
Heart failure	1551 (67.2%)	431 (73.3%)	1982	0.005
Diabetes	829 (35.8%)	171 (28.9%)	1000	0.002
Myocardial infarction	497 (21.4%)	92 (15.5%)	589	0.001
Valvular disease	646 (29.1%)	231 (41.8%)	877	<0.001
Atherosclerosis	863 (38.1%)	299 (51.2%)	1162	<0.001
Bleeding/anemia	335 (19.5%)	131 (29.1%)	466	<0.001
Laboratory parameters				
Left ventricular ejection fraction (EF, %)	45.9 ± 13.9	49.5 ± 10.9	—	<0.001
eGFR (ml/min)	54.8 ± 18.0	47.0 ± 14.8	—	<0.001
Creatinine (mg/dL)	1.23 ± 0.57	1.26 ± 0.44	—	<0.001
Hemoglobin (g/dL)	13.16 ± 1.99	12.16 ± 1.66	—	<0.001
NT-proBNP	4532 ± 6670	5612 ± 6767	—	<0.001
BMI (kg/m^2^)	29.37 ± 5.53	26.25 ± 4.18	—	<0.001
Treatment				
PCI	470 (20.3%)	83 (14.0%)	553	<0.001
Device (PM/ICD/CRT)	809 (34.9%)	289 (49.0%)	1098	<0.001
Statins	1391 (60.0%)	294 (49.9%)	1685	<0.001
ACE-I	1074 (46.4%)	232 (39.3%)	1306	0.002
MRA	1086 (46.9%)	164 (27.8%)	1250	<0.001
Diuretics	1739 (75.1%)	502 (84.9%)	2241	<0.001
Apixaban 2.5 mg	316 (13.6%)	241 (40.8%)	557	<0.001

Values are presented as *n* (%) for categorical variables and mean ± standard deviation (SD) for continuous variables. Comparisons between groups (<85 vs. ≥85 years) were performed using the Pearson chi-square test for categorical variables and the Mann–Whitney U test for continuous variables. The CHA_2_DS_2_-VA score represents thromboembolic risk assessment excluding sex category. Abbreviations: AF—atrial fibrillation; EF—ejection fraction; eGFR—estimated glomerular filtration rate; BMI—body mass index; ACE-I—angiotensin-converting enzyme inhibitors; MRA—mineralocorticoid receptor antagonists; PCI—percutaneous coronary intervention.

**Table 2:** Multivariable Logistic Regression in Patients ≥85 Years.

Therapy	Predictor	OR (95% CI)	*p*-Value
Apixaban 2.5 mg	CHA_2_DS_2_-VA	1.34 (1.11–1.64)	0.003
Apixaban 2.5 mg	eGFR	0.64 (0.47–0.89)	0.006
Dabigatran 150 mg	CHA_2_DS_2_-VA	0.35 (0.14–0.97)	0.038

Abbreviations: OR—odds ratio; CI—confidence interval; CHA_2_DS_2_-VA—stroke risk score; eGFR—estimated glomerular filtration rate.

## Data Availability

All data analyzed in this study are available from the corresponding author upon request.

## Supplementary Materials

The following supporting information can be downloaded at https://www.mdpi.com/article/10.3390/jcm15103806/s1, Table S1: Anticoagulation Patterns Across Major AF Registries and Trials; Table S2: Multivariable Logistic Regression Models for Antithrombotic Therapy in Patients Aged <85 Years.

## Abbreviations

AF	atrial fibrillation
ACE-I	angiotensin-converting enzyme inhibitor
ANAFIE	All Nippon AF In the Elderly Registry
APT	antiplatelet therapy
BMI	body mass index
CABG	coronary artery bypass grafting
CHA_2_DS_2_-VA	Congestive heart failure, Hypertension, Age, Diabetes, Stroke/transient ischemic attack, Vascular disease (sex category excluded)
CI	confidence interval
COMBINE-AF	Collaborative Analysis of Anticoagulation in Atrial Fibrillation
CRAFT	MultiCenter expeRience in AFib patients Treated with OAC
DOAC	direct oral anticoagulant
eGFR	estimated glomerular filtration rate
EF	ejection fraction
ELDERCARE-AF	Edoxaban Low-Dose for Elder Care Atrial Fibrillation
ENGAGE AF–TIMI 48	Effective Anticoagulation with Factor Xa Next Generation in Atrial Fibrillation–Thrombolysis in Myocardial Infarction 48
GLORIA-AF	Global Registry on Long-Term Oral Antithrombotic Treatment in Patients with Atrial Fibrillation
GARFIELD-AF	Global Anticoagulant Registry in the FIELD–Atrial Fibrillation
HAS-BLED	Hypertension, Abnormal renal/liver function, Stroke, Bleeding, Labile INR, Elderly, Drugs/alcohol
LMWH	low-molecular-weight heparin
MRA	mineralocorticoid receptor antagonist
NT-proBNP	N-terminal pro-B-type natriuretic peptide
NOAC	Non-vitamin K antagonist oral anticoagulant
OAC	oral anticoagulant
OR	odds ratio
PCI	percutaneous coronary intervention
POL-AF	Polish Atrial Fibrillation Registry
RCT	randomized controlled trial
SD	standard deviation
TIA	transient ischemic attack
VKA	vitamin K antagonist
