# Supplementary material for: What Drives Anticoagulant Selection in Patients Aged ≥85 Years with Atrial Fibrillation? Insights from the CRAFT Registry
Source: J Clin Med. 2026 May 15;15(10):3806. doi: 10.3390/jcm15103806 (PMC13207535; doi:10.3390/jcm15103806)
Supplement: Supplementary file 1 [file jcm-15-03806-s001.zip › jcm-4299244-supplementary.pdf]

Supplementary Table S1. Anticoagulation Patterns Across Major AF Registries and Trials.

Study	Study Type	Population	Mean Age (Year)	DOAC/NOAC (%)	VKA (%)	OAC Overall (%)	Ref.
ELDERCARE-AF	RCT	≥80 years, ineligible for standard OAC	86.6	50 (edoxaban)	0	50	[19]
FRAIL-AF	RCT	Frail elderly AF patients	~83	~50	~50	100	[24]
ANAFIE	Registry	≥75 years (Japan)	~81.6	~60–70	~20–30	>90	[17]
GLORIA-AF	Registry	Global AF registry	~70–72	~55–60	~20–25	~80–85	[25]
GARFIELD-AF	Registry	Global AF registry	~70	~30–40	~30–50	~70–80	[23]
Fushimi AF	Registry	Community-based registry (Japan)	~73–74	~50–60	~30–40	~80–90	[16]
POL-AF	Registry	Hospitalized AF patients (Poland)	~72.0	74.3	16	~90	[20]
CRAFT	Registry	≥85 years (Poland)	88.4	81.1	10.6	94.4	—

Values are presented as percentages unless otherwise indicated. OAC overall includes the use of DOACs and VKAs. Data are derived from published studies and may vary depending on study design and population.

Supplementary Table S2. Multivariable Logistic Regression Models for Antithrombotic Therapy in Patients Aged <85 Years.

Therapy	Model χ^2	Nagelkerke R^2	Main Predictors
Apixaban 2.5 mg	251.5	0.266	CHA_2DS_2-VA, sex, hypertension, diabetes, stroke/TIA, eGFR, bleeding
Apixaban 5 mg	24.6	0.037	Hypertension, CABG, eGFR, atherosclerosis
Rivaroxaban 15 mg	80.5	0.089	CHA_2DS_2-VA, hypertension, stroke/TIA, PCI, atherosclerosis, bleeding
Rivaroxaban 20 mg	160.1	0.165	CHA_2DS_2-VA, myocardial infarction, valvular disease, eGFR, bleeding
Dabigatran 110 mg	19.5	0.029	CHA_2DS_2-VA, bleeding
Dabigatran 150 mg	128.8	0.154	Sex, coronary artery disease, eGFR, atherosclerosis, bleeding

Model χ^2 represents the likelihood ratio test for overall model significance. Nagelkerke R^2 indicates the proportion of variance explained by the model. Only variables retained in the final stepwise logistic regression models (PIN 0.05, POUT 0.10) are presented. A p-value < 0.05 was considered statistically significant. Abbreviations: CHADS-VA – stroke risk score; PCI – percutaneous coronary intervention; CABG – coronary artery bypass grafting; eGFR – estimated glomerular filtration rate.
